# Exploring the relationship between burnout and mental health among village doctors: a study from Xun County, Henan Province

**DOI:** 10.3389/fpubh.2026.1747850

**Published:** 2026-01-27

**Authors:** Zihao Yang, Huashi Liu, Wenjun Song, Jinli Ren, Zixiu He, Chuansheng Wang, Ruiling Zhang

**Affiliations:** 1 Department of Psychological Counseling, The Second Affiliated Hospital of Henan Medical University (Henan Mental Hospital), Xinxiang, Henan, China; 2 School of Health, Fujian Medical University, Fuzhou, Fujian, China; 3 Henan Collaborative Innovation Center of Prevention and treatment of mental disorder, Xinxiang, Henan, China; 4 Brain Institute, Henan Academy of Innovations in Medical Science, Zhengzhou, Henan, China

**Keywords:** burnout, depersonalization, emotional exhaustion, mental health, village doctor

## Abstract

**Objective:**

Village doctors serve as the primary gatekeepers of healthcare in rural China. They often face heavy workloads, which may place them at increased risk of burnout and psychological distress. However, evidence regarding the association between different dimensions of burnout and mental health among this group remains limited. This study aimed to: (1) estimate the prevalence of burnout and probable mental health problems among village doctors in Xun County; and (2) examine the factors influencing village doctors’ mental health and its associations with different dimensions of burnout.

**Method:**

A cross-sectional survey was conducted among 769 village doctors in Xun County, Henan Province, China. Sociodemographic and job-related characteristics were collected. Burnout was assessed using the Maslach Burnout Inventory-General Survey, and mental health conditions was measured with the 12-item General Health Questionnaire. Chi-square tests, ANCOVA, and binary logistic regression were used to examine factors associated with mental health among village doctors.

**Result:**

Of the participants, 460 (59.8%) met criteria for burnout and 179 (23.3%) had mental health conditions. Significant differences between village doctors with and without mental health conditions were observed in family relationship, annual family income, annual income from public health services, physical exercise, and regular diet. ANCOVA revealed that after controlling for sociodemographic and job-related characteristics, there were significant group differences in emotional exhaustion (EE) and depersonalization (DP), but not in lack of personal accomplishment. Logistic regression indicated that poor family relationships (OR = 1.73, *p* = 0.043), EE (OR = 1.10, *p* < 0.001), and DP (OR = 1.17, *p* < 0.001) were positively associated with poor mental health, whereas having a higher annual family income (30,000–50,000 yuan vs. <30,000 yuan, OR = 0.62, *p* = 0.037) and receiving some income from public health services (<10,000 yuan vs. 0 yuan, OR = 0.58, *p* = 0.046) were inversely associated with poor mental health.

**Conclusion:**

Burnout and mental health conditions are highly prevalent among village doctors in Xun County. Both demographic/job characteristics and specific burnout dimensions (EE and DP) are associated with mental health conditions in this population. Strategies aimed at reducing EE and DP are necessary to improve mental health.

## 1 Introduction

In China, village doctors are primarily responsible for providing public health and basic medical services to rural residents (1). They also undertake other medical and health service-related work entrusted by the health and family planning administrative departments (2). They are permanently stationed at the bottom of the three-tier health service delivery system-village clinics-and serve as the “gatekeepers” of rural residents’ health (3). The stability and sustainable development of the village doctor workforce are therefore essential to ensuring equitable access to basic public health services for all rural residents (4). Village doctors shoulder the heavy task of disease prevention and control for rural residents in China, and burnout is highly prevalent among them (5, 6).

Burnout is a common phenomenon across various occupational settings (7). The International Classification of Diseases, 11th Revision (ICD-11) has recognized burnout (code QD85) as a syndrome resulting from chronic workplace stress (8). It is characterized by three dimensions: (1) emotional exhaustion (EE)-feelings of energy depletion and fatigue; (2) depersonalization (DP)-an increase in psychological distance from the job or an increase in negative or cynical feelings associated with the job; and (3) low professional accomplishment (LPA)-a sense of ineffectiveness and lack of accomplishment (8). A 2022 systematic review has indicated that the detection rate of burnout among Chinese village doctors was 59.8% (95% CI: 38.7–79.1) (9). This high rate has been largely attributed to poor working conditions and heavy workloads. The allocation of medical and health resources in rural areas of China remains inefficient and inequitable (10). Moreover, within the rural three-tier healthcare system, village clinics exhibit the lowest efficiency due to less capable medical staff, outdated facilities, and weaker economic support (10). Correspondingly, doctors at village clinics experience heavier work pressures (5) and had higher levels of burnout (11) compared with their counterparts in township health centers. Furthermore, limited health literacy and insufficient awareness of disease prevention among rural residents contribute to a higher prevalence of chronic conditions such as cardiovascular, cerebrovascular, and metabolic diseases compared with urban populations. This, in turn, further increases the workload and psychological strain on village doctors (12, 13).

Numerous empirical studies have shown that burnout is not only a workplace-specific problem, but is also strongly associated with an individual’s overall mental health. Chronic and severe burnout can significantly reduce well-being and job satisfaction (14), and is an important risk factor for depression (15), anxiety (15), and insomnia (16, 17). Moreover, burnout can reduce cognitive function in individuals by affecting levels of the arousal modulators (e.g., norepinephrine, dopamine) or even impairing synaptic connections in the prefrontal cortex (18), leading to decreased work efficiency. Furthermore, fatigue, body aches, and symptoms of the gastrointestinal system are particularly common in burnout (19), which may exacerbate psychological distress, creating a vicious cycle. Therefore, the serious consequences of burnout on an individual’s mental health not only bring great pain but also lead to a decline in productivity. Most studies on the relationship between burnout and mental health have focused on hospital-based physicians (20–22), while village doctors-who occupy a unique position in China’s rural healthcare system-remain understudied. Limited research has shown that mental health levels of village doctors in China are significantly lower than the Chinese population norm (23). Further exploration of the factors influencing Chinese village doctors’ mental health and its relationships with different dimensions of burnout holds important theoretical and practical significance.

Prior research has consistently shown that demographic characteristics (e.g., age, sex, social support) (24), lifestyle behaviors (e.g., physical exercise) (25), and socioeconomic status (e.g., household economic levels) (25) are associated with mental health. In addition, work-related attributes have been identified as important determinants of doctors’ mental health (26). Drawing on these empirical evidences, we selected 18 sociodemographic and job-related variables encompassing demographic characteristics, lifestyle behaviors, socioeconomic factors, and work-related attributes to comprehensively assess factors associated with the mental health of village doctors.

In China, village doctors operate within a largely standardized primary healthcare system, delivering both basic medical care and essential public health services under national policy frameworks (27). Henan Province is located in the North China Plain, with vast land suitable for agricultural production and human habitation (28). Due to this, Henan has extensive rural areas, a large agricultural population (28), and it represents a typical rural primary healthcare setting in China (29). Xun County, located in northern Henan, is a predominantly agricultural county (30). Therefore, this study investigated the prevalence of burnout and mental health conditions among village doctors working in village clinics in Xun County, Henan Province, China, and further examined the factors associated with village doctors’ mental health, with a particular focus on the three dimensions of burnout. The main hypothesis of this study is that each of the three dimensions of burnout is positively associated with poorer mental health among village doctors. The findings are expected to provide valuable theoretical insights into the occupational health of village doctors and, more importantly, offer empirical evidence to inform the development of targeted interventions and supportive policies. These efforts aim to alleviate burnout, promote mental well-being among village doctors, enhance the quality and stability of rural healthcare services, and ultimately advance health equity in rural China.

## 2 Method

### 2.1 Participants

This cross-sectional survey was conducted from May to July 2024 using a cluster sampling approach. The participants were village doctors in Xun County, Henan Province, China, who held valid qualification certificates as village doctors, physician assistants, or physicians, and were actively practicing in village clinics. According to government records, 1,181 village doctors were registered in the county, of whom approximately 300 had already left their positions. Ultimately, 830 practicing village doctors were surveyed in the field using the online questionnaire platform Wenjuanxing. A total of 769 valid questionnaires were returned, yielding a response rate of 92.6%. All participants provided informed consent prior to data collection. The study protocol was reviewed and approved by the Institutional Review Board of the Second Affiliated Hospital of Xinxiang Medical University, in accordance with the Declaration of Helsinki.

### 2.2 Measures

According to several empirical evidences (24–26), 18 sociodemographic and job-related characteristics were examined in this study to comprehensively assess factors associated with the mental health of village doctors, including age, sex, marital status, family relationships, physical exercise, regular diet, educational background, major, professional title, professional qualification, years of working, annual personal income, annual family income, annual income from medical services, annual income from agriculture, annual income from public health services, the situation of participating in farming work, the situation of participating in public health services.

Burnout was assessed by the 15-item Chinese version of the Maslach Burnout Inventory-General Survey (MBI-GS) (31). The MBI-GS is a 7-point Likert scale (0 = never to 6 = every day). The MBI-GS consists of 3 dimensions: EE (5 items), DP (4 items) and LPA (6 items). Higher scores on each of the 3 dimensions indicate higher risks of burnout. The cutoff scores for the three dimensions were EE ≥ 25, DP ≥ 11, and LPA ≥ 16. Based on these thresholds, burnout was categorized into four levels: “no burnout” was defined as scores below the threshold on all subscales; “mild burnout” was defined as scores equal to or above the threshold on one subscale; “moderate burnout” was defined as scores equal to or above the threshold on two subscales; “high burnout” was defined as scores equal to or above the threshold on all subscales. The MBI-GS showed excellent internal consistency, with Cronbach’s alpha coefficients of 0.950 for EE, 0.932 for DP, 0.902 for LPA, and 0.872 for the total scale.

The mental health of participants was assessed using the 12-item General Health Questionnaire (GHQ-12), which has shown satisfactory reliability and validity in Chinese populations (32, 33). The GHQ-12 is a 4-point Likert scale. Responses of “not at all” and “same as usual” are scored as 0, while “rather more than usual” and “much more than usual” are scored as 1. The total score ranges from 0 to 12, with higher scores indicating poorer mental health. A total score of ≥3 was used as the threshold for identifying potential mental health problems. The GHQ-12 demonstrated good internal consistency in the present sample, with a Cronbach’s alpha coefficient of 0.866.

### 2.3 Data analysis

All statistical analyses were performed using SPSS version 27.0 (IBM Corp., Armonk, NY, USA). To compare differences between village doctors with and without mental health conditions, categorical variables were analyzed using the chi-square test. Analysis of covariance (ANCOVA) was conducted to examine group differences in the three MBI-GS subscales after adjusting for sociodemographic and job-related characteristics. Finally, in the binary logistic regression analysis, we selected the variables that were significant in the chi-square test and ANCOVA as independent variables, including family relationships, annual family income, annual income from public health services, physical exercise, regular diet, EE, and DP, to examine the significance of their association with mental health. Before performing the binary logistic regression analysis, multicollinearity among independent variables was assessed using variance inflation factors (VIFs) and tolerance statistics. All variables met acceptable criteria (VIF < 10; tolerance > 0.1), indicating no significant multicollinearity. The significance level was set at *p* < 0.05, and multiple comparisons were adjusted using the Bonferroni correction.

## 3 Results

### 3.1 Sample characteristics

As shown in Table 1, a total of 18 sociodemographic and job-related characteristics of the village doctors were described. Most participants were male (62.0%). Regarding age distribution, 40 (5.2%) were aged 25–35 years, 209 (27.2%) were 36–45 years, 386 (50.2%) were 46–55 years, and 134 (17.4%) were older than 55 years. The majority of participants were married (94.3%), while only 5.7% were single, divorced, or widowed.

**Table 1:** Sociodemographic, job-related characteristics and burnout in village doctors with and without mental health conditions.

Variables	Total (*n*/%)	Without mental health conditions (n)	With mental health conditions (n)	*χ*^2^/*F*	df	*p*
Sex						
Male	477/62%	355	122	3.720	1	0.054
Female	292/38%	235	57			
Age (years)						
25 ~ 35	40/5.2%	32	8	3.654	3	0.301
36 ~ 45	209/27.2%	166	43			
46 ~ 55	386/50.2%	297	89			
>55	134/17.4%	95	39			
Marital status						
Single/Divorced/Widowed	44/5.7%	36	8	0.678	1	0.410
Married	725/94.3%	554	171			
Educational background						
Bachelor’s degree or above	106/13.8%	77	29	1.369	2	0.504
Junior college	178/23.1%	140	38			
High school or below	485/63.1%	373	112			
Family relationships						
Good	665/86.5%	528	137	19.709	1	**<0.001** ^***^
Bad	104/13.5%	62	42			
Major						
Western medicine	258/33.6%	199	59	0.734	2	0.693
General family medicine	399/51.9%	302	97			
Traditional Chinese medicine	112/14.6%	89	23			
Professional title						
Intermediate or above	77/10%	56	21	0.780	2	0.677
Primary	384/49.9%	297	87			
No title	308/40.1%	237	71			
Professional qualification						
Certified doctor	188/24.4%	143	45	1.739	3	0.628
Certified assistant doctor	153/19.9%	119	34			
General village doctor	173/22.5%	138	35			
Village doctor	255/33.2%	190	65			
Years of working (years)						
<5	33/4.3%	29	4	8.591	4	0.072
5 ~ 10	73/9.5%	62	11			
11 ~ 20	168/21.8%	132	36			
21 ~ 30	284/36.9%	216	68			
>30	211/27.4%	151	60			
Working form						
Full-time doctor	328/42.7%	255	73	5.084	2	0.079
Half-agricultural and half-medicine/Agricultural main medical auxiliary	336/43.7%	247	89			
Medical main agricultural auxiliary	105/13.7%	88	17			
The situation of participating in public health services						
Participation	633/82.3%	481	152	1.085	1	0.298
Non-participation	136/17.7%	109	27			
Annual personal income (CNY)						
<15,000	326/42.4%	248	78	0.179	2	0.914
15,000 ~ 20,000	241/31.3%	187	54			
>20,000	202/26.3%	155	47			
Annual family income (CNY)						
<30,000	382/49.7%	277	105	8.339	2	0.015^*^
30,000 ~ 50,000	269/35%	221	48			
>50,000	118/15.3%	92	26			
Annual income from medical services (CNY)						
<5,000	202/26.3%	145	57	4.706	3	0.195
5,000 ~ 10,000	255/33.2%	200	55			
10,001 ~ 300,000	251/32.6%	200	51			
>30,000	61/7.9%	45	16			
Annual income from public health services (CNY)						
0	60/7.8%	48	12	1.084	3	0.781
<20,000	473/61.5%	359	115			
20,001 ~ 400,000	186/24.2%	145	43			
>40,000	50/6.5%	38	9			
Annual income from agriculture (CNY)						
0	112/14.6%	75	37	8.157	2	0.017^*^
<10,000	490/63.7%	379	111			
≥10,000	167/21.7%	136	31			
Physical exercise						
Yes	569/74%	456	113	14.308	1	**<0.001** ^***^
No	200/26%	134	66			
Regular diet						
Yes	622/80.9%	499	123	22.346	1	**<0.001** ^***^
No	147/19.1%	91	56			
Burnout^a^						
EE		7.59 ± 5.92	14.66 ± 7.01	134.463	1	**<0.001** ^***^
DP		2.75 ± 3.63	7.84 ± 5.74	166.676	1	**<0.001** ^***^
PA		16.53 ± 10.10	16.20 ± 7.82	0.150	1	0.699

a Analysis of covariance (ANCOVA) to adjust for 18 Sociodemographic, job-related characteristics.

EE, emotional exhaustion; DP, depersonalization; PA, reduced professional accomplishment. *p < 0.05; **p < 0.01; ***p < 0.001. Values that are significant after Bonferroni correction for multiple comparisons were in bold.

### 3.2 Job burnout and mental health of village doctors

The overall mean score of the MBI-GS was 29.63 ± 13.64. The mean scores for the three subscales were as follows: EE was 9.24 ± 6.87, DP was 3.93 ± 4.73, and LPA was 16.45 ± 9.62. Based on their subscale scores, participants were classified into four levels of burnout: no burnout (*n* = 309, 40.2%), mild burnout (*n* = 424, 55.1%), moderate burnout (*n* = 31, 4.0%), and high burnout (n = 5, 0.7%). The total mean score of the GHQ-12 was 1.73 ± 2.61. According to the GHQ-12 cutoff score of ≥3, 179 participants (23.3%) were identified as having mental health conditions, whereas 590 participants (76.7%) were classified as having no mental health conditions.

### 3.3 Sociodemographic and job-related characteristics and burnout in village doctors with and without mental health conditions

Table 1 compares the sociodemographic and job-related characteristics, as well as burnout levels, between village doctors with and without mental health conditions. Significant group differences were observed in five sociodemographic and job-related characteristics: family relationships (*χ*^2^ = 19.709, *p* < 0.001), annual family income (*χ*^2^ = 8.339, *p* = 0.015), annual income from public health services (*χ*^2^ = 8. 157, *p* = 0.017), physical exercise (*χ*^2^ = 14.308, *p* < 0.001), and regular diet (*χ*^2^ = 22.346, *p* < 0.001). After adjusting for 18 sociodemographic and job-related covariates, ANCOVA results indicated significant group differences in EE (*F* = 134.463, *p* < 0.001) and DP (*F* = 166.676, *p* < 0.001), while the difference for LPA was not significant. Following Bonferroni correction (*p* < 0.05/21), only family relationships, physical exercise, regular diet, EE, and DP remained statistically significant.

**Figure 1:**
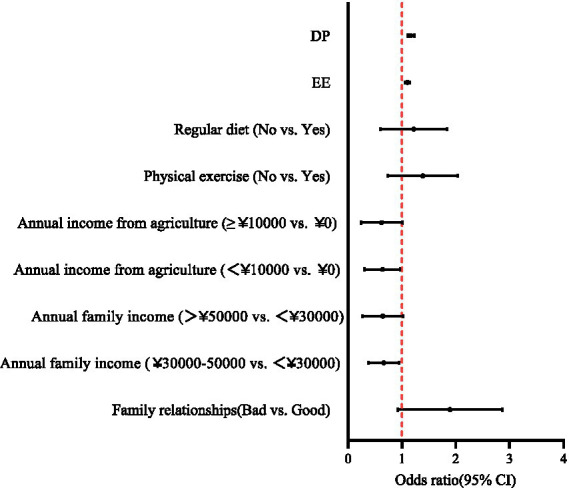
Forest plot of factors associated with mental health conditions among village doctors based on binary logistic regression analysis. EE, emotional exhaustion; DP, depersonalization.

**Figure 2:**
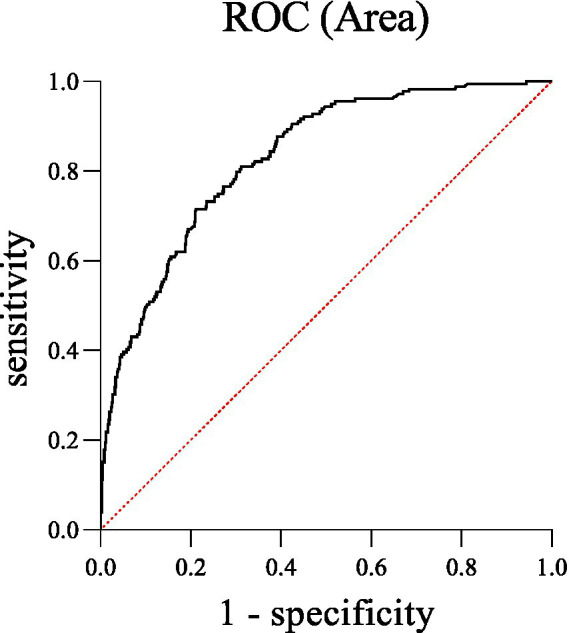
The discriminatory capacity of the combination of factors for distinguishing between village doctors with and without mental health conditions. The area under the ROC curve was 0.862.

Binary logistic regression analysis further showed that mental health conditions were independently associated with family relationships, annual family income, annual income from public health services, EE, and DP among village doctors (see Table 2 and Figure 1). Specifically, poor family relationship (OR = 1.729, *p* = 0.043), as well as higher levels of EE (OR = 1.103, *p* < 0.001) and DP (OR = 1.167, *p* < 0.001), were associated with higher odds of mental health conditions among village doctors. In contrast, having a higher annual family income (30,000–50,000 yuan vs. <30,000 yuan, OR = 0.621, *p* = 0.037) and receiving some income from public health services (<10,000 yuan vs. 0 yuan, OR = 0.582, *p* = 0.046) were associated with lower odds of mental health conditions. The model accounted for 35.4% of the total variance (Nagelkerke’s *R*^2^ = 0.354). In addition, ROC curve analysis yielded an AUC of 0.862, indicating strong discriminative ability of the regression model to distinguish village doctors with mental health conditions from those without (*p* < 0.0001, 95% CI = 0.798–0.862) (see Figure 2).

**Table 2:** Factors associated with mental health in village doctors (binary logistic regression analysis).

Variables	Beta	Wald 𝜒^2^	*p* value	Odds ratio (95%CI)
Family relationships				
Bad vs. Good	0.547	4.112	0.043*	1.729 (1.018, 2.935)
Annual family income (CNY)				
30,000–50,000 vs. <30,000	−0.476	4.354	0.037*	0.621 (0.397, 0.972)
>50,000 vs. <30,000	−0.560	3.224	0.073	0.571 (0.310, 1.053)
Annual income from agriculture (CNY)				
<10,000 vs.0	−0.542	3.988	0.046*	0.582 (0.342, 0.990)
≥10,000 vs. 0	−0.605	3.402	0.065	0.546 (0.287, 1.039)
Physical exercise				
No vs. Yes	0.253	1.060	0.303	1.287 (0.796, 2.082)
Regular diet				
No vs. Yes	0.113	0.178	0.673	1.119 (0.664, 1.887)
EE	0.098	28.584	<0.001^***^	1.103 (1.064, 1.144)
DP	0.154	35.083	<0.001^***^	1.167 (1.109, 1.228)

EE, emotional exhaustion; DP, depersonalization. *p < 0.05; **p < 0.01; ***p < 0.001.

## 4 Discussion

This study assessed burnout and mental health among 769 village doctors working in village clinics in Xun County, Henan Province, China, in 2024, and examined the associations between them. The main findings were as follows: (1) 59.8% of village doctors experienced varying degrees of burnout (primarily mild burnout), and 23.3% exhibited mental health conditions; (2) higher levels of EE and DP were significantly associated with poorer mental health, whereas LPA was not significantly related to mental health; and (3) beyond the three dimensions of burnout, poor family relationships, low annual family income, and low annual income from agriculture were also identified as independent risk factors for mental health conditions among village doctors.

Our study found that 59.8% of village doctors in Xun County experienced job burnout, with 7.8% suffering from moderate or high levels of burnout. In comparison, a survey of physicians in tertiary hospitals in Guangzhou reported a burnout prevalence of 45.7% (34), and a large-scale national survey showed that approximately 40% of radiologists in secondary and tertiary hospitals experienced burnout (35). During the COVID-19 epidemic, the prevalence of burnout was markedly higher among pulmonologists (61.7%) (36) and intensivists (82.1%) (37). These findings suggest substantial variation in burnout rates among physicians in urban China, with village doctors exhibiting a higher prevalence than most urban physicians—except for those in high-intensity specialties such as pulmonology and intensive care, whose work was directly and heavily affected by the pandemic (36). In addition, a cross-sectional study conducted in an eastern province of China during the COVID-19 epidemic reported that 53.47% of village doctors experienced burnout, with nearly 46% showing moderate or high levels (6). Furthermore, a 2022 systematic review that synthesized evidence from 20 studies involving 23,284 village doctors across almost all provinces in China found an overall burnout prevalence of 59.8% (95% CI: 38.7–79.1), with up to 20% experiencing moderate or high burnout (9). These findings indicate that the overall prevalence of burnout among village doctors has remained relatively stable in recent years, while the proportion of moderate and high burnout has substantially decreased. This improvement may be partly attributed to the end of the COVID-19 pandemic and the implementation of national “strengthening primary health care” policies—such as increased investment in grassroots medical infrastructure and workforce support—which have helped alleviate some of the occupational stress faced by village doctors (38). In other countries, the incidence of burnout among rural doctors is also high; however, differences in the questionnaire used and scoring methods make direct cross-country comparisons challenging. For instance, a survey using the Maslach Burnout Inventory-Human Service Survey (MBI-HSS) conducted in rural KwaZulu-Natal Province, South Africa, reported that 68.5% of village doctors experienced job burnout (22). Similarly, a study of general practitioners in rural Germany using the MBI-HSS reported that the incidence rates of EE, DP, and LPA were 50.6, 30.6, and 56.5%, respectively (39).

In this study, based on the GHQ-12 assessment, 23.3% of village doctors were found to have mental health conditions, indicating that nearly one in four experienced some degree of psychological distress—such as sleep disturbance, loss of confidence, or difficulty in decision-making. This prevalence is higher than that of the general Chinese population (18%) (40). A previous survey has reported that the prevalence of mental health conditions among primary care physicians in China was 29.2% during the acute phase of the COVID-19 pandemic (February 2020), which decreased to 21.8% 5 months later as the situation improved (41). Thus, the prevalence of mental health conditions among village doctors observed in this study is comparable to that of primary care physicians during the pandemic, suggesting that chronic stress may persist among village doctors even under routine, non-epidemic conditions.

Notably, this study found that among the three dimensions of burnout, each one-point increase in EE was associated with a 10.3% increase in the risk of mental health problems, and each one-point increase in DP was associated with a 16.7% increase in such risk, whereas LPA showed no significant effect. EE has been shown to correlate positively with elevated levels of salivary cortisol, consistent with the neurobiological mechanisms of the physiological stress response (42). Dysregulation of the hypothalamic–pituitary–adrenal (HPA) axis—reflected by abnormal cortisol secretion—is a well-established contributor to anxiety (43), depression (44), and other mental health disorders. Thus, EE should not be viewed merely as a subjective feeling of being “worn out,” but rather as a sustained physiological stress response that can adversely affect mental health.

DP demonstrated a stronger association with mental health problems than EE. This suggests that emotional detachment from patients and colleagues may serve as an even more serious warning sign for compromised psychological well-being among village doctors. Conceptually, DP has been described as a maladaptive emotion regulation strategy that involves emotional distancing and cynicism toward patients and colleagues (45). While such detachment may temporarily reduce emotional overload in high-demand settings, it is closely linked to dysfunctional coping patterns, including avoidance, withdrawal, and rumination (45). Over time, these patterns may erode social connectedness, impair interpersonal relationships, and exacerbate psychological distress (45), particularly in the socially embedded context of rural communities where professional and personal roles often overlap.

In contrast, the absence of a significant relationship between LPA and mental health suggests that feelings of inefficacy or low accomplishment, although unpleasant, may not be the principal drivers of mental health problems in this population. In resource-constrained rural settings, village doctors may normalize limited career advancement or recognition, whereas sustained emotional depletion and interpersonal disengagement exert a more direct impact on mental health.

Beyond burnout, mental health among village doctors was also shaped by sociodemographic and socioeconomic factors. In this study, the mental health status of village doctors varied significantly across several sociodemographic and job-related factors, including family relationships, annual family income, annual income from agriculture, regular physical exercise, and regular diet. Logistic regression analysis further revealed that good family relationships, higher annual family income, and higher agricultural income served as protective factors for the mental health of village doctors. From a conservation of resources perspective (46, 47), family support and financial security represent key external resources that help offset resource loss caused by chronic work demands. A supportive family can provide essential emotional relief, practical assistance, and a sense of belonging, all of which mitigate psychological distress. This aligns with extensive evidence indicating that strong social support functions as a critical buffer against stress and a key determinant of mental health (48, 49). Likewise, the protective role of economic stability-as reflected in higher family income and diversified income sources such as agriculture-underscores the importance of financial security. Financial strain is a well-documented chronic stressor that erodes cognitive and emotional resources (50). Having multiple income streams may reduce such strain and preserve the psychological capacity to manage work-related stress. Although regular exercise and a healthy diet were significantly associated with better mental health in univariate analyses, their effects were not retained in the multivariate model. This suggests that these lifestyle factors may exert indirect influences on mental health through pathways such as enhanced social support, improved economic stability, or reduced burnout, rather than functioning as independent predictors. Alternatively, individuals with greater financial means and stronger family support may be better positioned to maintain healthy lifestyles, including regular exercise and nutritious diets (51).

Interestingly, this study did not identify significant associations between any job-related characteristics-including educational background, medical major, professional title, professional qualification, years of working, working form, and the situation of participating in public health services-and mental health among village doctors. This finding may reflect the relatively constrained career trajectories within rural primary healthcare settings, where opportunities for professional advancement are limited. In the present study, 90% of village doctors in Xun County held only junior-level professional titles or no professional titles at all. Under such conditions, professional expectations may be uniformly low, and psychological distress may be driven less by formal professional status than by emotional demands and resource constraints.

These findings also have important policy and practice implications for occupational health and rural primary care systems. First, at the organizational level, targeted interventions should prioritize reducing emotional exhaustion and depersonalization, such as streamlining public health reporting requirements, and providing regular opportunities for psychological support within township health systems. Second, at the professional development level, the findings highlight the need to improve incentive mechanisms for village doctors, particularly by strengthening transparent and attainable pathways for professional title promotion, expanding access to continuing medical education, and optimizing job positions within township-level medical and health institutions (52). Third, at the socioeconomic level, policies should dynamically adjust the subsidy standards for village doctors through various channels and gradually improve their treatment levels (27).

Our study has several limitations. First, although the sample size was relatively large, all participants were recruited from a single county located in the North China Plain. Future research can conduct stratified sampling based on China’s rural development gradient (central plains, western mountainous areas, and eastern coastal areas). Second, while the GHQ-12 is a widely validated questionnaire for assessing psychological distress—including symptoms of anxiety, depression, and social dysfunction—it is relatively insensitive to somatic manifestations of mental health problems. As a result, the true prevalence of psychological distress among village doctors may be underestimated. Future studies should consider integrating multiple assessment tools, including additional standardized questionnaires and clinical diagnostic interviews, to enhance measurement precision. Third, the cross-sectional design of this study did not establish a clear “cause and effect” association between burnout and mental health conditions. Longitudinal or experimental studies are needed to clarify the directionality and temporal dynamics of this relationship.

## 5 Conclusion

In conclusion, this study identified a high prevalence of burnout (59.8%) and mental health conditions (23.3%) among village doctors in rural primary healthcare setting in China. Emotional exhaustion and depersonalization were significantly associated with poorer mental health, whereas reduced personal accomplishment showed no significant effect. Beyond occupational stressors, supportive family relationships and greater family and agricultural income emerged as important protective factors. These findings underscore the need for comprehensive, multi-level interventions that not only alleviate workplace stressors but also strengthen socioeconomic and familial support systems to promote and sustain the mental health of village doctors. This research highlights the critical importance of addressing both professional and personal resource dimensions to safeguard mental health, strengthen the stability of the rural healthcare workforce, and advance health equity in underserved regions.

## Data Availability

The original contributions presented in the study are included in the article/supplementary material, further inquiries can be directed to the corresponding authors.
